# Comparison of Carbapenem-Resistant *Klebsiella pneumoniae* Strains Causing Intestinal Colonization and Extraintestinal Infections: Clinical, Virulence, and Molecular Epidemiological Characteristics

**DOI:** 10.3389/fpubh.2021.783124

**Published:** 2021-12-03

**Authors:** Wenli Liao, Na Huang, Ying Zhang, Yao Sun, Tao Chen, Weiliang Zeng, Liqiong Chen, Hong Wen, Jianming Cao, Tieli Zhou

**Affiliations:** ^1^Department of Clinical Laboratory, The First Affiliated Hospital of Wenzhou Medical University, Wenzhou, China; ^2^Department of Laboratory, Yongzhou Central Hospital, Yongzhou, China; ^3^Department of Medical Lab Science, School of Laboratory Medicine and Life Science, Wenzhou Medical University, Wenzhou, China

**Keywords:** carbapenem-resistant, *Klebsiella pneumonia*, intestinal colonization, infection, virulence

## Abstract

Carbapenem-resistant *Klebsiella pneumonia* (CRKP) infections has become a concerning threat. However, knowledge regarding the characteristics of intestinal CRKP isolates is limited. This study aimed to investigate and compare the clinical, virulence and molecular epidemiological characteristics of intestinal colonization and extraintestinal infections CRKP strains. The clinical characteristics were investigated retrospectively. Polymerase chain reaction was used to investigate the capsular serotype, virulence genes and carbapenemase genes. Capsular polysaccharide quantification assay, serum resistance assay, biofilm formation assay, and infection model of *Galleria mellonella* larvae were performed to compare the virulence and pathogenicity. Besides, multilocus-sequence-typing (MLST) and pulsed-field-gel-electrophoresis (PFGE) were conducted to explore the homology of intestinal CRKP isolates. A total of 54 intestinal CRKP isolates were included. The main capsular serotypes were K14, K64, and K19. C-reactive protein and the proportion of ICU isolation of the infection group were significantly higher than that of the colonization group (*P* < 0.05). The carrier rates of various virulence genes of CRKP in the infection group were mostly higher than those in the colonization group, wherein the carrier rates of *peg-344* and *rmpA* were significantly different (*P* < 0.05). There was no significant difference in capsular polysaccharides, antiserum ability, biofilm formation ability between the two group (*P* > 0.05), but the lethality of the infection group to *Galleria mellonella* was significantly higher than that of the colonization group (*P* < 0.05). The MLST categorized the 54 isolates into 13 different sequence types. PFGE revealed that homology among the 54 CRKP strains was <80%. This study suggested that the CRKP strains in the infection group had higher virulence than those in the colonization group. The development of CRKP isolates colonizing in the intestine should be addressed in future clinical surveillance.

## Introduction

With the widespread use of carbapenems, the emergence of carbapenem-resistant *Klebsiella pneumoniae* (CRKP) has been increasingly reported worldwide (1–3). Numerous reports indicated that CRKP was considered as a serious threat to global health (4, 5).

The intestinal tract of hospitalized patients is an enormous repository of antibiotic-resistant bacteria (6). More seriously, antibiotic-resistant bacteria can not only colonize in the intestine tract, but also transfer to other tissues through intestinal metastasis, leading to serious extraintestinal infections (7), which will be an important risk factor for systemic infection in-hospital death of patients. However, studies have shown that not all *Klebsiella pneumoniae* (*K. pneumoniae*) colonized in the intestine could cause further infection (8), and the majority of patients with colonized pathogens did not suffer from infection for several years or even decades (9).

The highly pathogenic bacterium whose strong virulence has been attributed to its ability to resist the host's innate immunity and further infect the host invasively (10, 11). Studies have shown that high-virulence isolates usually have higher capsule content, antiserum ability, and carry a variety of virulence factors, including high-viscosity phenotypes, capsular serotypes, virulence genes, and high-virulence-related clone types, etc. (12, 13). The capsular polysaccharide around bacteria is a crucial virulence factor of *K. pneumoniae*, participating in the resistance to phagocytosis and serum bactericidal activity (14). Capsular polysaccharides are also closely related to the hyperviscosity phenotype, K1/K2 capsular serotype, and mucus phenotype regulatory gene A (*rmpA*) (7, 15). In addition, the adhesion of fimbriae is another vital virulence factor of *K. pneumoniae*. The gene *fimH* and *mrkD* promote bacterial adhesion to host tissues and organs by encoding type 1 and type 3 fimbriae of *K. pneumoniae*, leading to bacterial colonization and pathogenicity. Among them, *mrkD* can also promote the development of biofilms, which improves the bacteria's resistance to host defense and antibiotics (16).

This study retrospectively investigated the clinical and microbiological characteristics of patients with intestinal CRKP and compared the patients characteristics with CRKP asymptomatic intestinal colonization and extraintestinal infections. The virulence genes (*fimH, mrkD, peg-344, rmpA, rmpA*2, *icuA*) and virulence phenotype (capsular polysaccharides, antiserum ability, biofilm formation ability and the lethality of *Galleria mellonella* larvae) were studied to understand the virulence characteristics of the intestinal CRKP strains. Finally, the molecular epidemiological characteristics of the intestinal CRKP strains were analyzed by MLST and PFGE to provide a theoretical basis for the reasonable clinical management of the intestinal CRKP strains.

## Materials and Methods

### Bacterial Isolates

A total of 54 intestinal CRKP isolates were included in 2019, in The First Affiliated Hospital of Wenzhou Medical University in Wenzhou, China. All isolates were identified by the matrix-assisted laser desorption/ionization time-of-flight mass spectrometry (MALDI-TOF-MS) system. These isolates were stored at −80°C for further research. The intestinal CRKP isolates were divided into two groups by clinical data and homology analysis (MLST and PFGE). One group was the CRKP that caused only asymptomatic intestinal colonization (colonization group); the another group was the CRKP that caused extraintestinal infections after intestinal colonization (infection group). If the MLST and PFGE of the extraintestinal infection isolates were consistent with those isolated from the intestine, and the infection occurred later than the colonization of the intestinal strain, we thought it was caused by the colonization of the intestinal strain. The information on the strains isolated from extraintestinal infection sites was shown in Supplementary Table S1.

### Clinical Data Collection

The medical records were reviewed to integrally collect the data of the patients with intestinal CRKP strain during the study period. The study data included the following variables: demographic characteristics (gender and age), underlying or concomitant conditions (diabetes, malignant tumors, cardiovascular diseases, liver insufficiency, renal insufficiency, trauma, etc.), admission temperature, invasive procedures, use of hormones or immunosuppressants, inflammation indicators [white blood cell (WBC) count, C-reactive protein (CRP), procalcitonin (PCT)], admission to intensive care unit (ICU), and prognosis, etc.

### Antimicrobial Susceptibility Testing

The MICs of antimicrobial agents, including fosfomycin (FOS) and amikacin (AMK) were determined using the agar dilution method, and the MICs of tigecycline (TGC), colistin (COL) and ceftazidime/avibactam (CZA) were measured via the broth microdilution method (BMD). The results were interpreted by the latest guidelines published by the Clinical and Laboratory Standards Institute (CLSI; Pittsburgh, PA, USA) 2020 (17) and the European Committee on Antimicrobial Susceptibility Testing (EUCAST 2020) (http://www.eucast.org).

### Polymerase Chain Reaction (PCR) for Capsular Serotypes, Virulence Genes, and Carbapenemase Genes

DNA was extracted from CRKP strains. Subsequently, capsular serotype-specific gene (*wzi*), virulence genes (e.g., *fimH, mrkD, peg-344, rmpA, rmpA2* and *icuA*) and carbapenemase genes were amplified by PCR using specific primers as previously described (1, 18–20) (Table 1). The positive PCR products were verified by sequencing at Beijing Genomics Institute Technology Co. Ltd. (Shanghai, China). Nucleotide sequences were compared using BLAST (http://blast.ncbi.nlm.nih.gov/Blast.cgi).

**Table 1:** Primers used in this study.

**Genes**	**Primer sequence (5′-3′)**	**Annealing temperature (°C)**	**Size (bp)**
**Virulence genes**			
*fimH*	F: TGCTGCTGGGCTGGTCGATG	62	688
	R: GGGAGGGTGACGGTGACATC		
*mrkD*	F: CCACCAACTATTCCCTCGAA	43	226
	R: ATGGAACCCACATCGACATT		
*peg-344*	F: CTTGAAACTATCCCTCCAGTC	53	508
	R: CCAGCGAAAGAATAACCCC		
*rmpA*	F: GAGTAGTTAATAAATCAATAGCAAT	50	332
	R: CAGTAGGCATTGCAGCA		
*rmpA2*	F: GTGCAATAAGGATGTTACATTA	50	430
	R: GGATGCCCTCCTCCTG		
*icuA*	F: AATCAATGGCTATTCCCGCTG	59	239
	R: CGCTTCACTTCTTTCACTGACAGG		
**Capsular serotype**			
*wzi*	F: GTGCCGCGAGCGCTTTCTATCTTGGT ATTCC	58	580
	R: GAGAGCCACTGGTTCCAGAA(C/T)TT (C/G)ACCGC		
**Carbapenemase genes**			
*bla*_KPC−2_	F: CGCCAATTTGTTGCTGAAGG	55	341
	CATAGTCATTTGCCGTGCCA		
*bla*_NDM_	F: GGTTTGGCGATCTGGTTTTC R: CGGAATGGCTCACGATC	52	621
	F: GGTTTGGCGATCTGGTTTTC R: CGGAATGGCTCACGATC		
*bla*_VIM_	F: GGTCTCATTGTCCGTGATGGTGATGAG	50	271
	R: CTCGATGAGAGTCCTTCTAGAG		
*bla*_IMP_	F: CATGGTTTGGTGGTTCTTGT	50	488
	R: ATAATTTGGCGGACTTTGGC		
*bla*_OXA−48_	F: CGCATCTTGTTGTCCAAGTG	52	1,012
	R: TCGAGCATCAGCATTTTGTC		
**Housekeeping genes**			
*gapA*	F: TGAAATATGACTCCACTCACGG	60	450
	R: CTTCAGAAGCGGCTTTGATGGCTT		
*infB*	F: CTCGCTGCTGGACTATATTCG	50	318
	R: CGCTTTCAGCTCAAGAACTTC		
*mdh*	F: CCCAACTCGCTTCAGGTTCAG	50	477
	R: CCGTTTTTCCCCAGCAGCAG		
*pgi*	F: GAGAAAAACCTGCCTGTACTGCTGGC	50	432
	R: CGCGCCACGCTTTATAGCGGTTAAT		
*phoE*	F: ACCTACCGCAACACCGACTTCTTCGG	50	420
	R: TGATCAGAACTGGTAGGTGAT		
*rpoB*	F: GGCGAAATGGCWGAGAACCA	50	501
	R: GAGTCTTCGAAGTTGTAACC		
*tonB*	F: CTTTATACCTCGGTACATCAGGTT	45	414
	R: ATTCGCCGGCTGRGCRGAGAG		

### Capsular Polysaccharide Quantification Assay

Capsular polysaccharide quantification assay was performed as described previously (21, 22), with minor modifications. Briefly, 500 μL of cultured bacteria suspensions were resuspended in phosphate-buffered saline (PBS) and adjusted to 10^8^ CFU/mL, and a 1.2 mL sodium tetraborate in sulfuric acid was added in the resuspensions that were later placed in the ice bath and incubated for 5 min at 100°C, and then place on ice for 10 min. A 20 μL volume of 1.5 mg/mL m-hydroxyldiphenyl was then added and mixed. After a 5 min incubation at room temperature, the absorbance at 590 nm was measured. The glucuronic acid content was determined from a standard curve of glucuronic acid and expressed as μg/10^8^ CFU. Results were presented as the mean and standard deviation (Mean ± SD) of the data of three independent experiments.

### Serum Resistance Assay

Serum resistance assay was performed as described previously (23), with minor modifications. Briefly, overnight bacterial cultures were diluted 1:100 into 10 mL of fresh Luria-Bertani (LB) medium and incubated until the bacterial suspension equaled to an OD_600_ of 0.5. Then, a 1 mL aliquot of the culture was washed with PBS and resuspended in 1 mL of PBS. Next, 100 μL of the bacterial suspension was mixed with 300 μL of normal human serum (NHS) and PBS. Bacteria-PBS mix was regarded as control. After mixing, the serum-bacteria and PBS-bacteria suspensions were incubated at 37°C for 3 h. To calculate the serum bactericidal effect, a 100 μL aliquot was taken from each suspension and 10-fold serial dilutions were plated onto LB plates. The serum bactericidal effect was expressed as the ratio of the CFUs in the serum-bacteria suspension to the CFUs in the PBS-bacteria suspension. All experiments were performed in triplicate, and results were expressed as survival percentage.

### Biofilm Formation Assay

The biofilm formation assay was measured using the method of Wilksch et al. (24). Briefly, clinical isolates were grown to logarithmic phase in LB broth and diluted 1:100 into fresh LB broth. A total of 200 μL of each dilution were added to a 96-well polystyrene microtiter plate and blank controls were set at the same time, and each strain set three duplicate wells. Then, the plate was incubated at 37°C for 24 h. Planktonic cells were removed, and the wells were washed three times with sterile water and then stained with 200 μL 0.1% crystal violet for 10 min and rinsed three times with sterile water. Stained biofilms were solubilized with 95% (V/V) ethanol and quantified by measuring the OD_595_. Each sample was measured in triplicates and averages of absorbance values were used for analysis.

### Infection Model of *Galleria mellonella* Larvae

The virulence of *K. pneumoniae* was evaluated by the *G. mellonella* larvae infection model, as previously described (25). Briefly, serial dilutions of *K. pneumoniae* (10^4^, 10^5^, 10^6^, 10^7^ CFU/mL) were prepared in PBS. Subsequently, a 10 μL of each dilution was injected into the last left proleg. Larvae injected with 10 μL PBS were used as the control group. Ten larvae weighing between 200 and 250 mg were randomly selected for each dilution. The larvae were incubated at 37°C in the dark and observed at 24 h intervals in a week. Larvae were considered dead when they repeatedly failed to respond to physical stimuli. The primary outcome for the *G. mellonella* model was rapidity and extent of mortality of larvae assessed with Kaplan-Meier analysis and log-rank test.

### Multi-Locus Sequence Typing (MLST) and Pulsed-Field Gel Electrophoresis (PFGE)

In this study, seven housekeeping genes of *K. pneumoniae* (*rpoB, gapA, phoE, mdh, tonB, pgi*, and *infB*) were amplified by polymerase chain reaction (PCR) and sequenced. The genotypes of all isolates were identified according to the protocol provided on the designated website. The alleles and STs were assigned according to the Pasteur Institute multilocus sequence typing online database for *K. pneumoniae* (https://bigsdb.pasteur.fr/klebsiella/klebsiella.html).

PFGE was performed on our strains based on our previously described method (1), with only minor modifications. Genomic DNA was extracted from the *K. pneumoniae* strains, and digested with *Xba I* (Takara 1093A, Japan) for 2 h. PFGE was performed using a CHEF-Mapper XA PFGE system (Bio-Rad, USA) for 18 h with a molecular weight of 30–600 kb and a switch time of 6–36 s. DNA fingerprints were then revealed by Ethidium-bromide staining. The GelDoc XR gel imaging system (Bio-Rad, USA) was used to visualize stripe patterns.

### Statistical Analysis

All statistical analyses were performed using SPSS 22.0 software (SPSS Inc., Chicago, IL, USA). The Chi-Square test and Fisher's exact test were used to compare categorical variables. Student's *t*-test and Mann-Whitney *U* test were used to compare continuous variables with and without normal distribution, respectively. *P*-value <0.05 was considered statistically significant. All tests were two-tailed.

## Results

### Clinical Characteristics

The results showed that the median age of patients with intestinal CRKP was 59-year-old, and males accounted for 61.1%. 51 cases (94.4%) suffered from concomitant diseases, including 12 (22.2%) diabetes, 15 (27.8%) cardiovascular and cerebrovascular diseases, 14 (25.9%) renal insufficiency, 24 (44.4%) liver insufficiency, 5 (9.3%) trauma and so on. Inflammatory indicators include leukocytosis, C-reactive protein, and procalcitonin, all of which were increased. 45 (83.3%) have received invasive procedures, and 37 (68.5%) have been admitted to the ICU. Intestinal CRKP strains were mainly from ICU (29.6%), neurosurgery (16.7%) and respiratory medicine (14.8%). Among the 54 patients with intestinal CRKP strains, 13 caused extraintestinal infections after intestinal colonization (defined as the infection group), and 41 were only intestinal asymptomatic colonization (defined as the colonization group). C-reactive protein and the proportion of ICU isolates in the patients of the infected group were significantly higher than those in the colonization group (*P* < 0.05). The clinical data of 54 patients were shown in Table 2.

**Table 2:** Clinical characteristics of 54 patients with intestinal colonization CRKP.

**Clinical characteristic**	**Total (*n* = 54) *n* (%)**	**Infection group (*n* = 13) *n* (%)**	**Colonization group (*n* = 41) *n* (%)**	***P*-value**
Age, years, median (interquartile range)	59 (48, 72)	59 (47, 83.5)	59 (48, 69)	0.504
**Gender**				
Male	33 (61.1)	10 (76.9)	23 (56.1)	0.180
Female	21 (38.9)	3 (23.1)	18 (43.9)	0.180
**Department**				
ICU	16 (29.6)	8 (61.5)	8 (19.5)	**0.011**
Neurosurgery	9 (16.7)	1 (7.7)	8 (19.5)	0.569
Respiratory medicine	8 (14.8)	1 (7.7)	7 (17.1)	0.703
Other	21 (38.9)	3 (23.1)	18 (43.9)	0.180
**Underlying conditions**				
Diabetes	12 (22.2)	2 (15.4)	10 (24.4)	0.766
Malignant tumor	4 (7.4)	1 (7.7)	3 (7.3)	1.000
Cardiovascular diseases	15 (27.8)	3 (23.1)	12 (29.3)	0.937
Renal insufficiency	14 (25.9)	6 (46.2)	8 (19.5)	0.122
Liver insufficiency	24 (44.4)	5 (38.5)	19 (46.3)	0.618
Trauma	5 (9.3)	2 (15.4)	3 (7.3)	0.745
Admission temperature (°C)	37.3 (36.8, 37.8)	37.5 (36.7, 37.8)	37.2 (36.9, 37.8)	0.731
Intrusive operation	45 (83.3)	11 (84.6)	34 (82.9)	1.000
Use hormones and/or immunosuppressive	13 (24.1)	0	13 (31.7)	0.050
**Laboratory examination**				
WBC count, × 10^9^/L	10.16 (7.07, 13.70)	9.52 (6.13, 9.52)	10.28 (7.37, 14.28)	0.473
CRP count, U/L	36.60 (21.25, 77.80)	65.40 (35.95, 90.00)	33.85 (15.03, 64.85)	**0.022**
PCT, ng/ml	0.202 (0.200, 0.423)	0.181 (0.131, 0.454)	0.212 (0.104, 0.390)	0.688
Admission to ICU	37 (68.5)	9 (69.2)	28 (68.3)	1.000
**Prognosis**				
Effective	35 (64.8)	5 (38.5)	30 (73.2)	0.051
Ineffective	19 (35.2)	8 (61.5)	11 (26.8)	0.051
**Clinical outcomes**				
Length of stay in hospital, days	33 (19.0, 45.0)	38 (21.5, 49)	30 (17.6, 40.3)	0.282
Time from admission to colonization	10 (5.5, 19)	14 (6, 25.5)	10 (5.3, 13.8)	0.469
Time from colonization to infection		9 (6.5, 14.5)		

*ICU, Intensive Care Unit; WBC, White blood cell; CRP, C-reactive protein; PCT, Procalcitonin. Bold values in the P-value column indicated statistical significance*.

### Antimicrobial Susceptibility Testing

The resistance rates of 54 CRKP strains to TGC, COL, CAZ/AVI, AMK, and FOS were 13, 7.4, 16.7, 40.7, and 55.6%, respectively. The susceptible rates of 54 CRKP strains to TGC, COL, CAZ/AVI, AMK, and FOS were 42.6, 92.6, 83.3, 57.4, and 25.9%, respectively. There were higher susceptible rates to colistin and ceftazidime/avibactam, and a higher intermediate rates (69.2%) to tigecycline (Table 3).

**Table 3:** Resistance profile of CRKP strains colonized in the intestine.

**Antibiotics**	**Total (*****n*** **= 54)** ***n*** **(%)**			**Infection group (*****n*** **= 13)** ***n*** **(%)**			**Colonization group (*****n*** **= 41)** ***n*** **(%)**		
	**R**	**I**	**S**	**R**	**I**	**S**	**R**	**I**	**S**
TGC	7 (13.0)	24 (44.4)	23 (42.6)	2 (15.4)	9 (69.2)	2 (15.4)	5 (12.2)	15 (36.6)	21 (51.2)
COL	4 (7.4)	–	50 (92.6)	2 (15.4)	–	11 (84.6)	2 (7.4)	–	39 (95.1)
AMK	22 (40.7)	1 (1.9)	31 (57.4)	8 (61.5)	0	5 (38.5)	14 (34.1)	1 (2.5)	26 (63.4)
CZA	9 (16.7)	–	45 (83.3)	2 (15.4)	–	11 (84.6)	7 (17.1)	–	34 (82.9)
FOS	30 (55.6)	10 (18.5)	14 (25.9)	5 (38.5)	5 (38.5)	3 (25.9)	25 (61.0)	5 (12.2)	11 (26.8)

*TGC, Tigecycline; COL, Colistin; AMK, Amikacin; CZA, Ceftazidime/Avibactam; FOS, Fosfomycin; R, Resistant; I, Intermediate; S, Susceptible; –, No Intermediate*.

### Carbapenemase Genes

**Figure 1:**
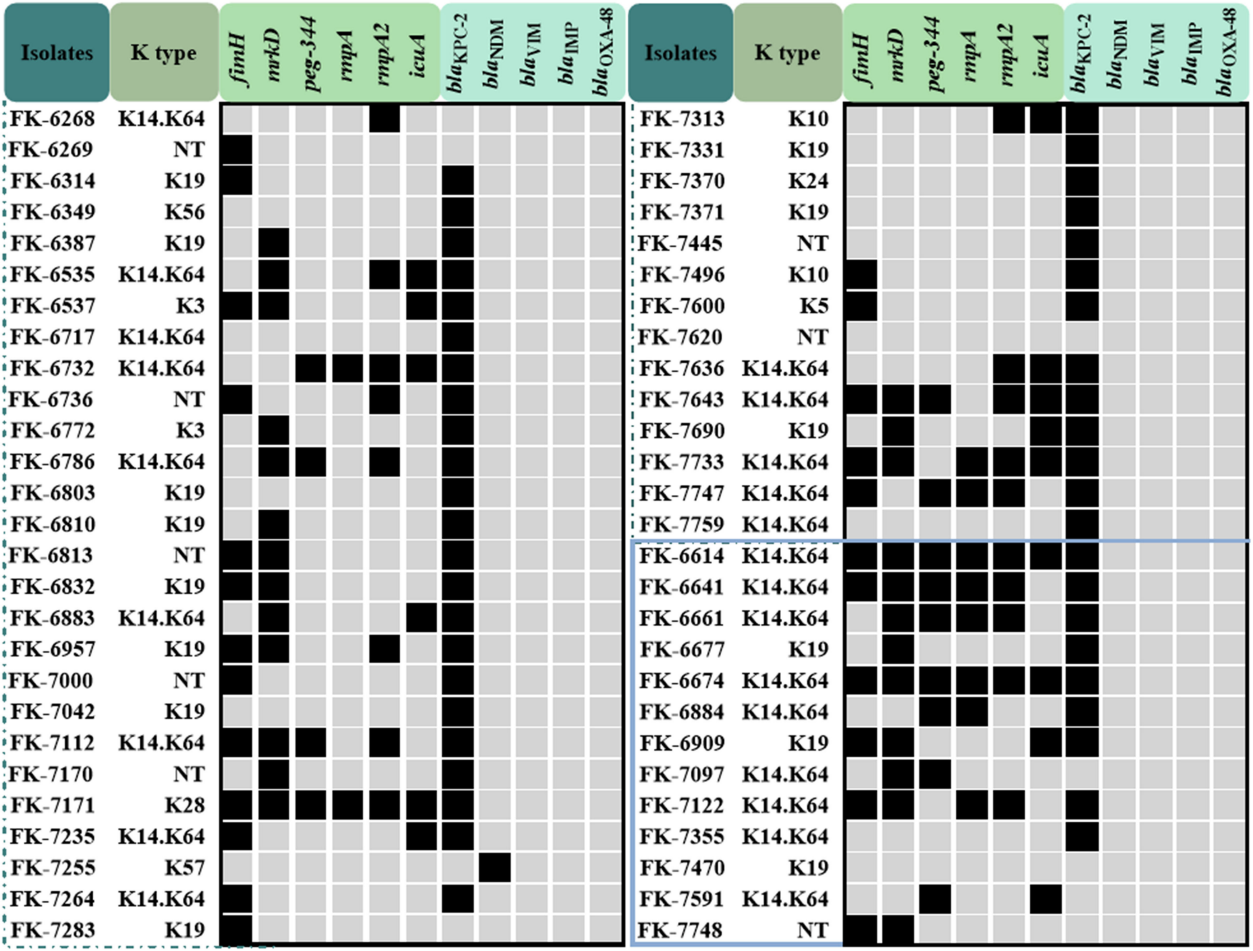
The capsular serotype, virulence genes and carbapenemase genes map of the colonization group and the infection group CRKP strains. The strains in the blue box represent the infection group strains, the others are the colonization group strains.

83.3% (45/54) of CRKP strains carried the *bla*_KPC−2_ gene, and *bla*_KPC−2_ was the dominant carbapenemase gene no matter in the infection group (69.2%, 9/13) or the colonization group (87.8%, 36/41). In addition, a strain carrying *bla*_NDM_ was also detected in the colonization group strains. *bla*_VIM_, *bla*_IMP_ and *bla*_OXA−48_ were not detected in all strains. The results of carbapenemase genes of 54 CRKP strains were shown in Figure 1.

### Capsular Serotypes and Virulence Genes

In the infection group, K14 and K64 serotypes accounted for 69.2% (9/13), followed by K19 serotypes accounting for 23.1% (3/13), and one strain could not be typed; in the colonization group strains, K14 and K64 serotypes accounted for 42.6% (23/41), K19 serotype accounted for 25.9% (14/41), and 8 strains could not be typed. The carrier rates of *fimH, mrkD, peg-344, rmpA, rmpA2*, and *icuA* of 54 CRKP strains were 44.4, 88.9, 24.1, 20.4, 33.3, and 27.8%, respectively. The carrier rate of virulence genes in the infection group was higher than that of the colonization group except for *mrkD*, and the difference of *peg-344* and *rmpA* was significant (*P* < 0.05). The results of the capsular serotype and virulence genes of 54 CRKP strains were shown in Figure 1 and Table 4.

**Table 4:** The capsular serotype and virulence genes of the infection group and the colonization group.

**Genes**	**Total** **(*n* = 54) *n* (%)**	**Infection group (*n* = 13) *n* (%)**	**Colonization group (*n* = 41) *n* (%)**	***P*-value**
**Capsular serotype**				
K3	2 (3.7)	0	2 (4.9)	1.000
K5	1 (1.9)	0	1 (2.4)	1.000
K10	2 (3.7)	0	2 (4.9)	1.000
K14, K64	23 (42.6)	9 (69.2)	14 (34.1)	**0.026**
K19	14 (25.9)	3 (23.1)	11 (26.8)	1.000
K24	1 (1.9)	0	1 (2.4)	1.000
K28	1 (1.9)	0	1 (2.4)	1.000
K56	1 (1.9)	0	1 (2.4)	1.000
K57	1 (1.9)	0	1 (2.4)	1.000
non-type	8 (14.8)	1 (7.7)	7 (17.1)	0.703
**Virulence genes**				
*fimH*	24 (44.4)	6 (46.2)	18 (43.9)	0.887
*mrkD*	48 (88.9)	9 (69.2)	39 (95.1)	**0.037**
*peg-344*	13 (24.1)	7 (53.8)	6 (14.6)	**0.012**
*rmpA*	11 (20.4)	6 (46.2)	5 (12.2)	**0.024**
*rmpA2*	18 (33.3)	5 (38.5)	13 (31.7)	0.910
*icuA*	15 (27.8)	5 (38.5)	10 (24.4)	0.528

*Bold values in the P-value column indicated statistical significance*.

### The Virulence Phenotype

**Figure 2:**
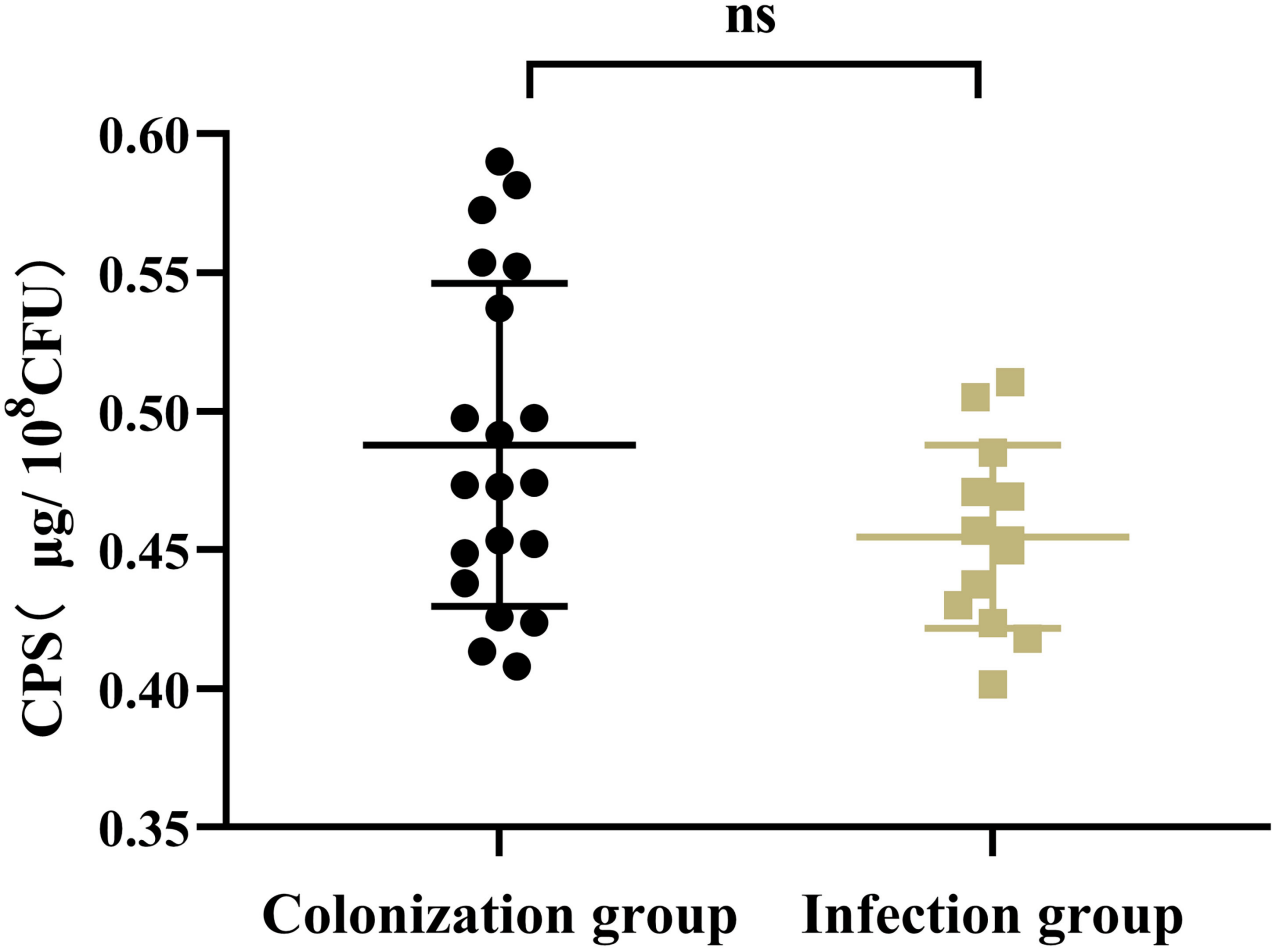
Capsular polysaccharide content of intestinal CRKP strains. ns, no significant difference.

**Figure 3:**
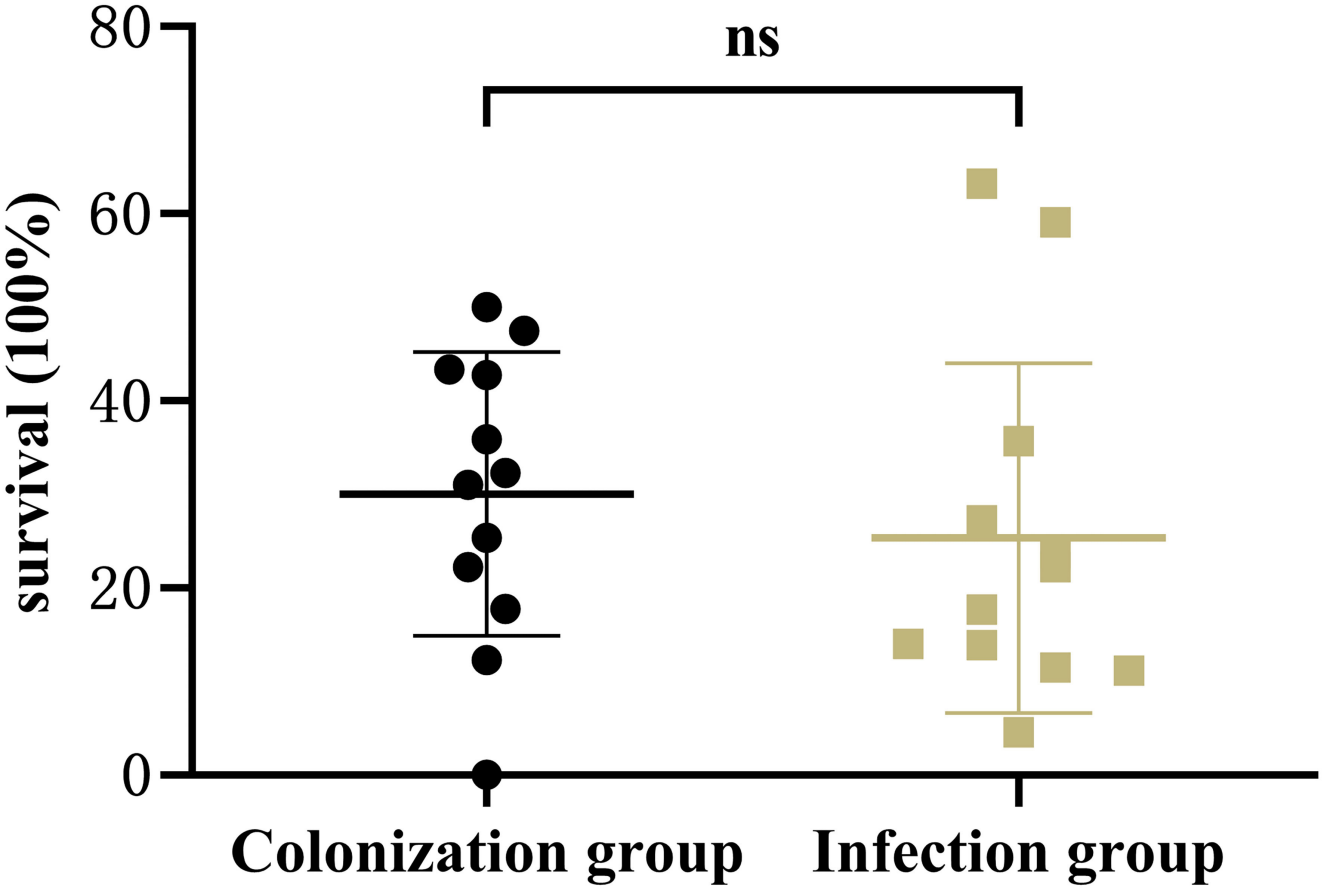
Antiserum ability of intestinal CRKP strains. ns, no significant difference.

**Figure 4:**
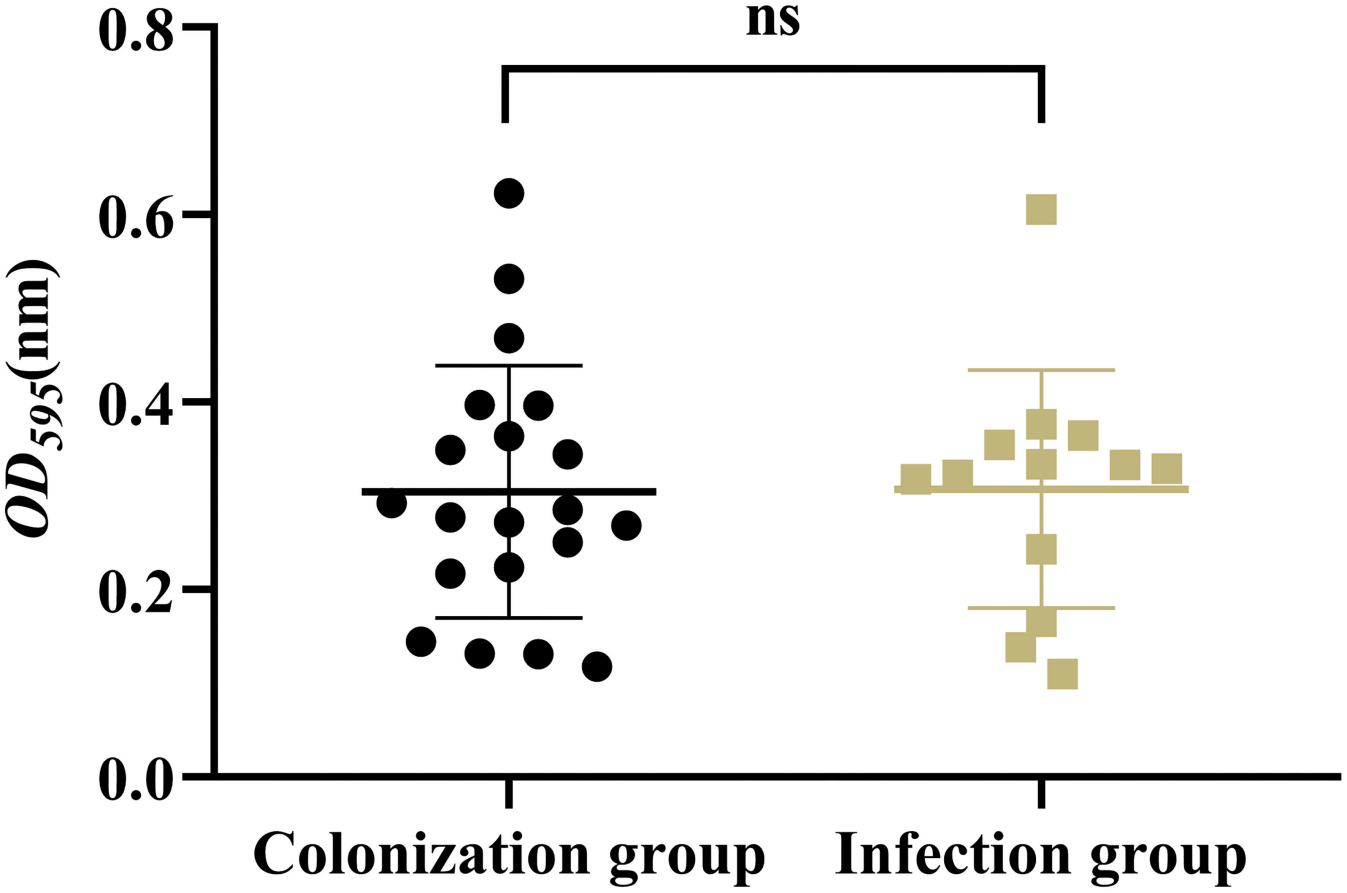
Biofilm-forming ability of intestinal CRKP strains. ns, no significant difference.

**Figure 5:**
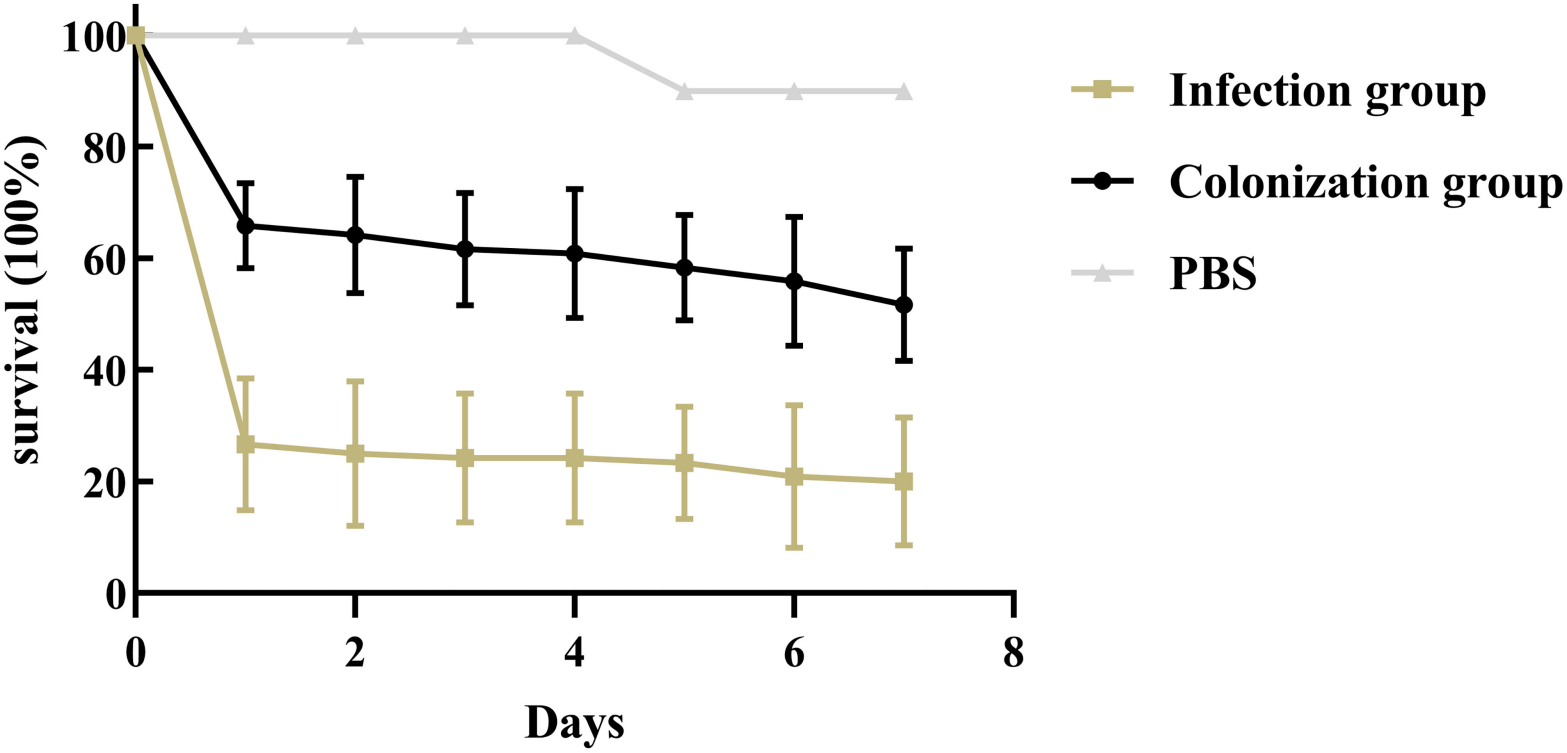
Infection model of *Galleria mellonella* larvae. Survival of *Galleria mellonella* larvae infected by intestinal CRKP strains.

Results showed that there was no significant difference in the capsular polysaccharide content, antiserum effect and biofilm formation between colonization group and infection group strains (*P* > 0.05) (Figures 2–4); The survival situations of *G. mellonella* larvae infected with CRKP strain were shown in Figure 5. The results showed that the lethality rate of *G. mellonella* larvae in the infection group was significantly higher than that of the colonization group (*P* < 0.05). The lethality rate of the two groups was significantly higher than that of the PBS control group (*P* < 0.05).

### Multi-Locus Sequence Typing (MLST) and Pulsed-Field Gel Electrophoresis (PFGE)

**Figure 6:**
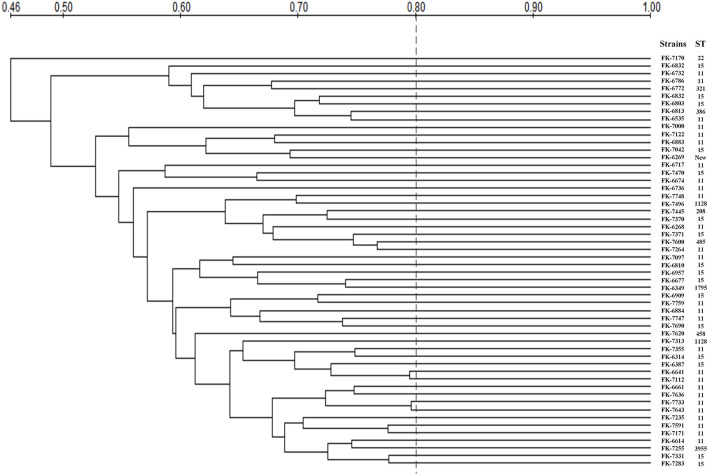
PFGE profile and MLST of 54 intestinal CRKP strains. New, New ST.

The MLST and PFGE results of 54 strains were shown in Table 5 and Figure 6. MLST showed that the 54 CRKP strains belonged to 13 different ST, among which ST11 and ST15 were the most concentrated, accounting for 27 (50%) and 15 (27.78%), respectively. In infection group, there were only two ST types: ST11 and ST15. However, in the colonization group, there were a few other rare ST types besides ST11 and ST15, such as ST22, ST208, ST321, ST386, ST458, ST485, ST896, ST1128, ST1795, ST395. In addition, a new kind of ST was observed in infection group. PFGE showed that 54 CRKP strains belonged to different pulse types, and their similarities were all <80%. The strains displayed low phylogeny in both the colonization group and infection group, indicating that no clonal transmission has been found.

**Table 5:** Genotypes and distribution of *K. pneumoniae* isolates.

**ST**	**Total** **(*n* = 54) *n* (%)**	**Infection group (*n* = 13) *n* (%)**	**Colonization group (*n* = 41) *n* (%)**
11	27 (50)	10 (76.92)	17 (41.46)
15	15 (27.78)	3 (23.08)	12 (29.27)
22	1 (1.85)	0	1 (2.44)
208	1 (1.85)	0	1 (2.44)
321	1 (1.85)	0	1 (2.44)
386	1 (1.85)	0	1 (2.44)
458	1 (1.85)	0	1 (2.44)
485	1 (1.85)	0	1 (2.44)
896	1 (1.85)	0	1 (2.44)
1,128	2 (3.70)	0	2 (4.88)
1,795	1 (1.85)	0	1 (2.44)
3,955	1 (1.85)	0	1 (2.44)
New	1 (1.85)	0	1 (2.44)

*New, New ST*.

## Discussion

In recent years, the detection rate of CRKP strains has been reported to be increasing (26), which has brought great challenges to clinical treatment. In order to understand the difference between intestinal colonization and causing-extraintestinal infections CRKP strains, we investigated and compared the clinical and microbiological characteristics of intestinal CRKP isolates.

In this study, patients with intestinal CRKP were mainly middle-aged and elderly men, consistent with the conclusion of Chen et al. (27), most of which were accompanied by diabetes, liver and kidney insufficiency, and cardiovascular diseases. The CRP of patients in the infection group was significantly higher than that of the colonization group (*P* < 0.05), which suggested that the CRP could be used as an early warning factor for secondary infection after colonization. Among patients with intestinal colonized CRKP, 45 patients (83.3%) have received invasive procedures, and 37 patients (68.5%) have been admitted to ICU, which suggested that invasive procedures and ICU admission may be the cause of CRKP colonization, which is consistent with the conclusions of previous reports (27, 28). The number of patients in ICU, neurosurgery, and respiratory was much more, and the isolation rate of the infection group strains in the ICU was significantly higher than that in the colonization group (*P* < 0.05), suggesting that for patients in ICU, neurosurgery and respiratory should undergo routine CRKP screening when they are admitted to the hospital to prevent and control CRKP infection. The results of clinical data showed that the median time from admission to intestinal CRKP colonization of patients was 10-day, and the median time from intestinal CRKP colonization to infection was 9-day. It is necessary to take preventive and control measures as early as possible for the prevention and treatment of infection.

Our study showed that the most prevalent carbapenemase gene was *bla*_KPC−2_ among CRKP isolates, which is consistent with previous studies (29, 30). There were 8 strains that have not been detected in any of *bla*_KPC−2_, *bla*_NDM_, *bla*_VIM_, *bla*_IMP_, or *bla*_OXA−48_, which may be caused by other resistance mechanisms, such as extended-spectrum β-lactamase, efflux pumps and porin mutations (31). Due to the production of carbapenemase and extended-spectrum β-lactamase (32), CRKP strains are resistant to general antibacterial drugs such as carbapenem and cephalosporins. At present, only a few drugs, such as colistin, tigecycline, some aminoglycoside drugs, and the recently approved ceftazidime/avibactam, still have good antibacterial activity against CRKP (33–35). The antimicrobial susceptibility testing showed that the intestinal CRKP strains had a high resistance rate to amikacin and fosfomycin, and the resistance rate to tigecycline and ceftazidime/avibactam were relatively low, accounting for 13 and 16.7%, respectively, which are lower than the results of Park and Zhou et al. (36, 37). Among all antibiotics tested, the most susceptible on CRKP strains was colistin, which was lower than the resistance rate reported by Rojas et al. (38). Colistin is still a good choice for the treatment of CRKP infection. However, the side effects such as nephrotoxicity and neurotoxicity should also be taken seriously. In severe CRKP infection, colistin is often recommended to combined with other antibiotics (such as tigecycline, meropenem, gentamicin, or fosfomycin, etc.) (39).

The virulence level of *K. pneumoniae* is closely related to the invasion and defense. High virulence helps the bacteria resist the host's innate immunity and invasively infect the host (10, 11). High viscosity is one of the most extrusive characteristics of highly virulent *K. pneumoniae*. Although the high viscosity of the bacteria is related to high virulence, it is not a necessary condition that high-viscosity *K. pneumoniae* is not equal to high-virulent *K. pneumoniae*. Multilocus sequence typing (MLST) and capsular antigen serotypes have also been used to identify high-virulent *K. pneumoniae*, even some special STs (such as ST23, ST65, and ST86) and capsular serotypes (such as K1 and K2) are closely related to high-virulent *K. pneumoniae*, but these genotypes and serotypes can also exist in classic *K. pneumoniae*. In 54 researched strains, no matter in the colonization group or the infection group, no K1, K2 capsular serotypes and ST23, ST65, and ST86 strains were found, but this cannot completely rule out high-virulence *K. pneumoniae*. Research (40) reported that there were also high-virulent strains of *K. pneumoniae* in ST11, and the intestinal CRKP strains in this study are mainly concentrated in ST11, especially in the infection group, ST11 strains account for a larger proportion.

Studies (18) have reported that *peg-344, iucA, rmpA*, and *rmpA*2 and other virulence genes can be used as candidate biomarkers for identifying *K. pneumonia* with high virulence, which showed the accuracy was >0.95. These specific genes mediate the increasing production of siderophores and capsular polysaccharides, thereby enhancing the high viscosity and virulence of *K. pneumoniae*. Although such laboratory indicators have high specificity, they are not only caused by high-virulent *K. pneumoniae*, but also exists in classic *K. pneumoniae*. Our results showed that the carrier rate of virulence genes in the infection group was higher than that in the colonization group, and the difference of *peg-344* and *rmpA* was significant (*P* < 0.05). Nevertheless, there was no significant difference in the production of capsular polysaccharides and antiserum killing ability of strains in infection group and colonization group. This may be closely related to the content of capsular polysaccharides in the strains. Studies have shown that capsular polysaccharides can hinder the reaction of bacteria with serum antibodies and other components (13, 21). Fimbriae adhesion is another prominent virulence factor for the increased virulence of *K. pneumoniae*. *fimH* and *mrkD* mediate adhesion by encoding *K. pneumoniae* type 1 and type 3 fimbriae, respectively, and promote bacterial adhesion to host tissues and organs, leading to bacterial colonization and pathogenicity. In addition, *mrkD* can also promote the development of biofilms, the physical barrier formed can hinder the attack of phagocytic killer cells and enzymes on bacteria, and increase the invasion of bacteria to the host and lead to antibiotics-resistance (16). However, our results showed that the carrier rate of *mrkD* in the infection group was significantly lower than that in the colonization group, but there was no significant difference in their biofilm formation ability. In order to explore the *in vivo* virulence and pathogenicity of the intestinal CRKP strains, we established the CRKP infection model of *G. mellonella* larvae. The results showed that the lethality rate of the infection group was significantly higher than that of the colonization group (*P* < 0.05), and the lethality rate of the two groups of strains was significantly higher than that of the PBS control group. It proved that the CRKP strains of infection group have higher virulence and stronger pathogenicity. Clinicians need to pay attention to patients with intestinal colonized CRKP, and take preventive and inspection measures in time to minimize the occurrence of highly virulent *K. pneumoniae* infections.

In this study, 54 intestinal CRKP strains belonged to different pulse types, suggesting that the strains had low homology. MLST showed that the intestinal colonized CRKP strains belonged to 13 ST types, among which ST11 and ST15 were the most concentrated, accounting for 27 (44.4%) and 15 (27.8%) respectively, and all the infection group strains were ST11 and ST15. The most common CRKP strains are ST11 and ST258 in the world, as well ST11 is the main ST in China, and ST258 is the most common in the United States (41, 42). ST11 is a single locus variant (*tonB*) of ST258, suggesting a close relationship between them. According to reports (42), ST11 is the main ST type of KPC-producing *K. pneumoniae*. Therefore, it can be speculated that *K. pneumoniae* of some STs (such as ST11 and ST258) are good carriers of drug-resistant plasmids and promote the spread of drug resistance. Some studies believed that (43) the ICEKp258.2 element of ST258 and ST11 strains can encode one or more factors, which may contribute to the persistence and spread of the strain. Specifically, ICEKp258.2 can encode IV fimbriae, which enables the strain to adhere to the surface of a variety of materials and/or improve its ability to exchange genetic information, thereby contributing to the persistence of the strain and stability (43). Therefore, we need to pay attention to the spread of ST11 *K. pneumoniae* between patients. In addition, ST15 is a single locus variant of ST14 (*infB*), which may represent the spread in the midwest of the United States, but it has now spread in China (42). ST15 and ST14 may belong to another potential clonal complex that may spread in China. According to reports (44), most of the isolates carrying *bla*_OXA−232_ belonged to ST15, therefore, attention should be paid to the prevalence of ST15 in the intestinal CRKP. The dominant clone group is relatively easy to develop carbapenem resistance, so molecular epidemiological monitoring can effectively control the occurrence and spread of drug-resistant bacterial infections in hospitalized patients.

The limitation of this study is the limited number of strains. This study showed that the CRKP strains in the infection group had higher virulence than those in the colonization group, which was mainly manifested in the carrier rates of various virulence genes and the mortality to *Galleria mellonella*, except capsular polysaccharide, antiserum ability and biofilm forming ability, which needs to be expanded in the future for further research.

## Conclusions

Our results suggested that the CRKP strains in the infection group had higher virulence than those in the colonization group. The development of CRKP isolates colonizing in the intestine should be addressed in future clinical surveillance.

## Data Availability Statement

The original contributions presented in the study are included in the article/Supplementary Material, further inquiries can be directed to the corresponding author/s.

## Ethics Statement

All the investigation protocols in this study were approved by the Ethics Committee of The First Affiliated Hospital of Wenzhou Medical University. Informed consent was waived because this study with an observational approach had mainly focused on bacteria and involved no interventions to the patients.

## Author Contributions

WL conducted the experiments, analyzed the data, and wrote the manuscript. YZ and TC participated in experiments. WZ and LC participated in writing. NH participated in the analysis of results. JC and TZ helped design the study. YS designed the study and corrected the manuscript. HW guided the revision of the manuscript. All authors have read and approved the manuscript.

## Funding

We thank the National Natural Science Foundation of China (no. 81971986), the Health Department of Zhejiang Province of the People's Republic of China (no. 2019KY098), and the Planned Science and Technology Project of Wenzhou (no. Y2020974) for providing financial funding.

## Conflict of Interest

The authors declare that the research was conducted in the absence of any commercial or financial relationships that could be construed as a potential conflict of interest.

## Publisher's Note

All claims expressed in this article are solely those of the authors and do not necessarily represent those of their affiliated organizations, or those of the publisher, the editors and the reviewers. Any product that may be evaluated in this article, or claim that may be made by its manufacturer, is not guaranteed or endorsed by the publisher.
