# Supplementary material for: Comparison of Carbapenem-Resistant Klebsiella pneumoniae Strains Causing Intestinal Colonization and Extraintestinal Infections: Clinical, Virulence, and Molecular Epidemiological Characteristics
Source: Front Public Health. 2021 Dec 3;9:783124. doi: 10.3389/fpubh.2021.783124 (PMC8678278; doi:10.3389/fpubh.2021.783124)
Supplement: Supplementary file 1 [file Table_1.docx]

**Table S1. The information on the strains isolated from extraintestinal infection sites.**

| **Intestinal  strains ^a^** | **Isolate date** | **Extraintestinal infection strains ^b^** | **Isolate  date** | **Specimen source** |
| --- | --- | --- | --- | --- |
| FK-6614 | 23 April | FK-6668 | 4 May | Blood |
|  |  | FK-6749 | 19 May | Sputum |
|  |  | FK-6745 | 20 May | Urine |
|  |  | FK-6768 | 23 May | Fecal |
|  |  | FK-6809 | 2 June | Fecal |
| FK-6641 | 29 April | FK-6712 | 15 May | Sputum |
| FK-6661 | 2 May | FK-6712 | 11 May | Sputum |
| FK-6677 | 5 May | FK-6729 | 13 May | Sputum |
| FK-6674 | 6 May | FK-6739 | 18 May | Sputum |
| FK-6884 | 16 June | FK-6880 | 18 June | Sputum |
|  |  | FK-6914 | 21 June | Blood |
|  |  | FK-6907 | 22 June | Urine |
| FK-6909 | 21 June | FK-6979 | 5 July | Sputum |
| FK-7097 | 31 July | FK-7123 | 6 August | Sputum |
| FK-7122 | 6 August | FK-7227 | 28 August | Alveolar lavage fluid |
|  |  | FK-7286 | 5 September | Wound |
| FK-7355 | 19 September | FK-7407 | 4 October | Sputum |
| FK-7470 | 18 October | FK-7504 | 26 October | Sputum |
| FK-7591 | 13 November | FK-7626 | 20 November | Sputum |
|  |  | FK-7662 | 28 November | Alveolar lavage |
|  |  | FK-7682 | 2 December | Blood |
|  |  | FK-7689 | 4 December | Urine |
| FK-7748 |  | FK-7752 |  | Sputum |

^a^: The strains of the infection group, all of which were isolated from the intestine.
^b^: The strains isolated from the extraintestinal infection site of patients in the infection group.
